# *Paliurus ramosissimus* Leaf Extract Inhibits Adipocyte Differentiation In Vitro and In Vivo High-Fat Diet-Induced Obesity Through PPARγ Suppression

**DOI:** 10.3390/ph18101515

**Published:** 2025-10-10

**Authors:** Shin-Hye Kim, Tae Hyun Son, Hye-Lim Shin, Dongsoo Kim, Gwang Hun Park, Jeong Won Seo, Hwan-Gyu Kim, Sik-Won Choi

**Affiliations:** 1 Forest Biomaterials Research Center, National Institute of Forest Science (NIFoS), Jinju 52817, Republic of Korea; black7a@korea.kr (S.-H.K.); snoopyegg@korea.kr (T.H.S.); hlims0901@korea.kr (H.-L.S.); skimds@korea.kr (D.K.); knight01@korea.kr (J.W.S.); 2 Department of Biological Sciences, Jeonbuk National University, Jeonju 54896, Republic of Korea; hgkim@jbnu.ac.kr; 3 Research Planning and Coordination Division, National Institute of Forest Science (NIFoS), Seoul 02455, Republic of Korea; ppkh0230@korea.kr

**Keywords:** obesity, *Paliurus ramosissimus*, adipogenesis, PPARγ, HFD-induced obesity

## Abstract

**Background/Objectives**: Obesity, defined by the excessive accumulation of adipose tissue, is associated with an increased risk of type 2 diabetes and metabolic dysfunction-associated steatotic liver disease (MASLD). Obesity treatments based on natural products are receiving increasing attention as viable alternatives to conventional treatments. **Methods**: To investigate the anti-obesity effects of *Paliurus ramosissimus* leaf extract (PRLE) in vitro and in vivo, we conducted studies using 3T3-L1 pre-adipocytes. The in vivo studies used high-fat diet (HFD)-fed C57BL/6 mice. PRLE effects were assessed through Oil Red O staining, RT-qPCR, Western blot, and morphological analysis of adipose tissue. **Results:** PRLE significantly reduced lipid accumulation in 3T3-L1 cells without cytotoxicity. PRLE treatment decreased mRNA expression of adipogenic genes (PPARγ, C/EBPα, FABP4, and leptin) and protein levels of adipogenesis-related markers. In HFD-fed mice, PRLE administration significantly reduced body weight gain (*p* < 0.001), decreased adipose tissue mass, and diminished the weight and size of white adipose tissue. **Conclusions:** PRLE exhibits anti-obesity effects both in vitro and in vivo, suggesting its potential as a therapeutic agent for obesity prevention.

## 1. Introduction

Obesity is one of the most important societal and individual health challenges globally. The prevalence of obesity escalated during the COVID-19 pandemic, since children, adolescents, and young adults during this period increased their food consumption while engaging in less physical activity during this period [1]. During the lockdown, 41.7% of adolescents in Palestine reported experiencing weight gain, which they attributed to increased consumption of fried foods, sweets, sugary drinks, and dairy products [2]. This markedly elevated the risk of cardiovascular diseases and type 2 diabetes, and it also increased the risks of poor bone health and certain types of cancer. Furthermore, obesity adversely affects quality of life, impacting sleep and mobility [3]. According to the WHO, one in eight individuals will be classified as obese by 2022. In 2024, there were 35 million children under five years old who were classified as overweight. When comparing the global obesity rates with those of 1990, the prevalence among adults has nearly doubled, while the prevalence among adolescents has quadrupled [3]. Overweight and obesity are generally the consequences of unhealthy lifestyle choices, including the consumption of excessive high-calorie foods, frequent eating, and snacking post-meals [4]. The positive energy balance leading to the accumulation of adipose tissue contributes to weight gain and obesity. The surplus energy is converted into lipids through adipogenesis, with these lipids being stored in adipocytes. During adipogenesis, mesenchymal stem cells differentiate into adipocytes. Consequently, obesity is typically characterized by an increase in the size and/or number of adipocytes within adipose tissue [5]. Despite the increasing incidence of obesity, few effective treatments are currently available. This underscores the urgent need for further research into obesity treatment options.

Obesity treatment currently can be broadly categorized into three types of pharmacological agents: those that modulate appetite through central nervous system mechanisms, those that stimulate thermogenesis in either the central or peripheral systems, and those that reduce gastrointestinal absorption. However, some of these drugs have significant adverse side effects, including nausea, vomiting, depression, and insomnia [6]. Consequently, there is a growing demand for alternative obesity treatments, such as plant-based anti-obesity remedies. Medicinal herbs are increasingly regarded as viable alternatives to conventional treatments for the management and prevention of various diseases, primarily due to their safety during long-term treatment and the absence of notable side effects [7]. Indeed, when compared with pharmaceutical interventions, natural-based anti-obesity treatments are perceived as having fewer or negligible side effects, which has contributed to their rising popularity. A growing number of individuals are seeking safe and effective anti-obesity treatments based on natural ingredients [8]. Therefore, the utilization of natural products constitutes a promising approach for addressing the current limited options available for the treatment of obesity.

The *Rhamnaceae* family has a wide global distribution, comprising approximately 55 genera and 950 species [9]. In Korea, there are 14 species across seven genera, including *Berchemia*, *Hovenia*, *Paliurus*, *Rhamnella*, *Rhamnus*, *Sageretia*, and *Zizyphus* [10]. To date, phylogenetic and chemical compositional studies have been conducted on the *Rhamnaceae* family, along with recent investigations into its bioactivities, including antioxidant and anti-inflammatory properties. Specifically, research has been carried out on the anti-inflammation properties of 13 species from the *Rhamnaceae* family in Korea [11]. And indigenous Rhamnaceae plants in Korea show antioxidant and immunomodulatory effects. Research on extracts from 13 species’ leaves, branches, and fruits revealed high antioxidant activity and NO production inhibition, particularly in *Berchemia berchemiifolia*, *Sageretia thea*, and *Paliurus ramosissimus* [12]. However, there is a significant gap in research focusing on the anti-obesity effects of this family. Studies on the pharmaceutical properties of *Paliurus ramosissimus* extracts are limited. This study aimed to investigate whether a 70% ethanol extract of *Paliurus ramosissimus* leaves (PRLE) would have anti-obesity effects, both in vitro and in vivo. Therefore, the present study reports the anti-obesity effects of *Paliurus ramosissimus* leaf extracts (PRLE) and its potential mechanism.

## 2. Results

### 2.1. PRLE Inhibits Lipid Accumulation in 3T3-L1 Adipocytes

**Figure 1:**
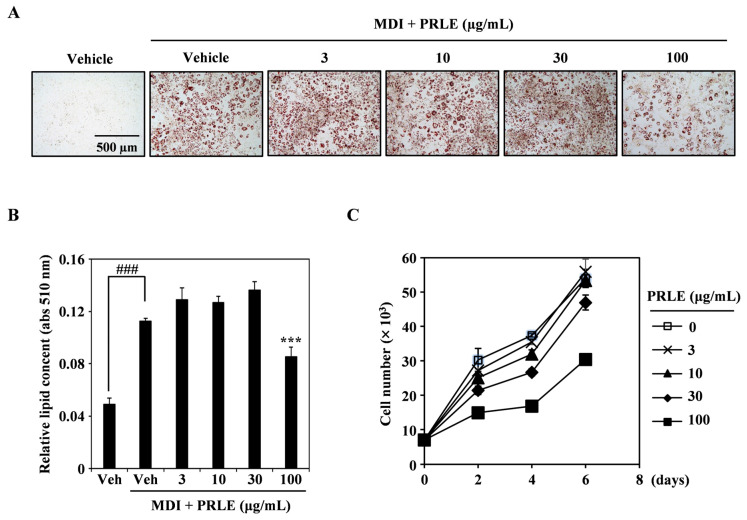
**The *Paliurus ramosissimus* Leaf (PRLE) extract significantly reduced lipid accumulation in adipocytes derived from 3T3-L1 Cells.** (**A**) A representative image illustrating the differentiation of 3T3-L1 pre-adipocytes following treatment with MDI and PRLE. (**B**) Statistical analysis of the images in panel (**A**), indicating ### *p* < 0.001 when compared to the control and *** *p* < 0.001 when compared to the MDI-treated control. (**C**) Assessment of 3T3-L1 cell proliferation in the presence of different PRLE concentrations.

To investigate whether PRLE inhibits adipogenesis, we stimulated 3T3-L1 cells with medium containing MDI (a cocktail of methylisobutylxanthine, dexamethasone, and insulin) to induce their differentiation into adipocytes and various concentrations of PRLE. The results showed that PRLE inhibited adipogenesis in a dose-dependent manner (Figure 1A). Relative lipid contents showed that MDI induced adipocyte differentiation, while PRLE significantly reduced the lipid content of the adipocytes (Figure 1B). Furthermore, cell proliferation in the presence of PRLE was higher than that at baseline on day zero. Notably, as the PRLE concentration in the medium increased, cell proliferation declined, but with no dateable cytotoxicity (Figure 1C). Therefore, it can be concluded that PRLE effectively inhibits lipid accumulation in 3T3-L1 cells without eliciting cytotoxicity.

### 2.2. PRLE Decreases the Expression of Molecular Markers Associated with Adipose Differentiation of 3T3-L1 Cells

**Figure 2:**
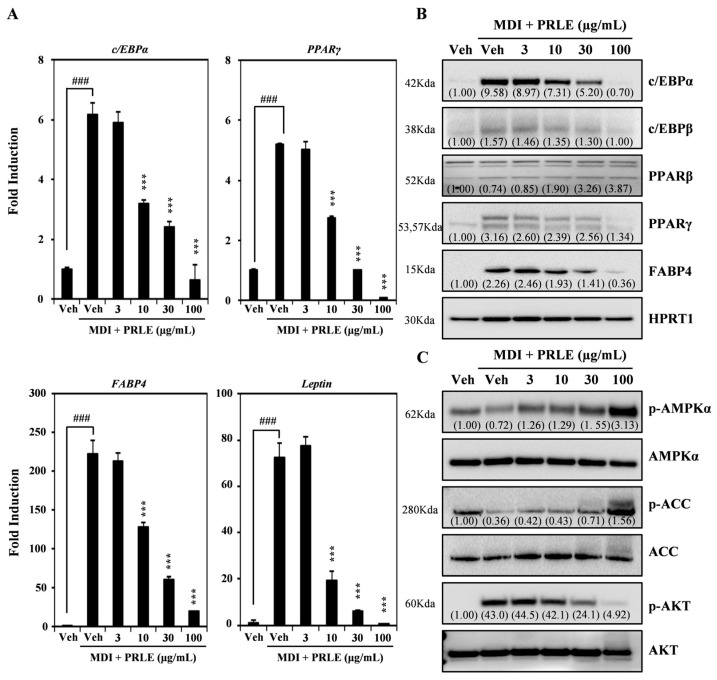
**PRLE inhibits MDI-induced adipogenic gene expression in 3T3-L1 Cells**. (**A**) On day 6 of differentiation, the mRNA expression of transcription factors involved in adipocyte differentiation was assessed using qRT-PCR in 3T3-L1 cells stimulated with either vehicle or PRLE at 3, 10, 30, and 100 μg/mL. HPRT1 served as the internal control. ### *p* < 0.001 (versus control), *** *p* < 0.001 (versus MDI-treated control). (**B**) Following day 6 of differentiation, the impact of PRLE on the protein expression levels of adipogenesis-related markers was evaluated through Western blot analysis, with HPRT1 utilized as the internal control. (**C**) The protein expression of signaling pathway components was analyzed via Western blot in 3T3-L1 cells stimulated with either vehicle or PRLE at concentrations of 3, 10, 30, and 100 μg/mL. After 2 h of serum starvation, the 3T3-L1 cells were pre-treated for 1 h with either vehicle or PRLE and then treated with MDI. Following MDI treatment for 30 min, the 3T3-L1 cells were harvested. Total AMPK α, ACC, and AKT were used as controls. A representative result of three independent experiments with similar results is presented.

To elucidate how PRLE affects adipogenesis, we employed RT-qPCR to assess the expression of molecular markers of adipocyte differentiation. The results showed that PRLE dose-dependently inhibited mRNA expression of transcription factors associated with adipogenesis, including C/EBPα and PPARγ. Additionally, increasing concentrations of PRLE suppressed the mRNA expression of adipogenesis-related molecular markers, such as FABP4 and Leptin (Figure 2A). Western blot analysis further corroborated these results, revealing a concentration-dependent decrease in the expression levels of C/EBPα, C/EBPβ, PPARγ, and FABP4 following PRLE treatment. Notably, the expression of PPARβ was unaffected by PRLE treatment (Figure 2B). Furthermore, we observed that PRLE increased the phosphorylation levels of AMPK and ACC. Conversely, the AKT phosphorylation induced by MDI was decreased after PRLE treatment (Figure 2C). Together, the results indicate that PRLE treatment suppresses adipogenesis by downregulating the expression of markers related to adipocyte differentiation, probably by altering the activity of AMPK and AKT signaling pathways.

### 2.3. PRLE Inhibits Body Weight Gain and Reduces Body Fat in HFD-Fed Mice

**Figure 3:**
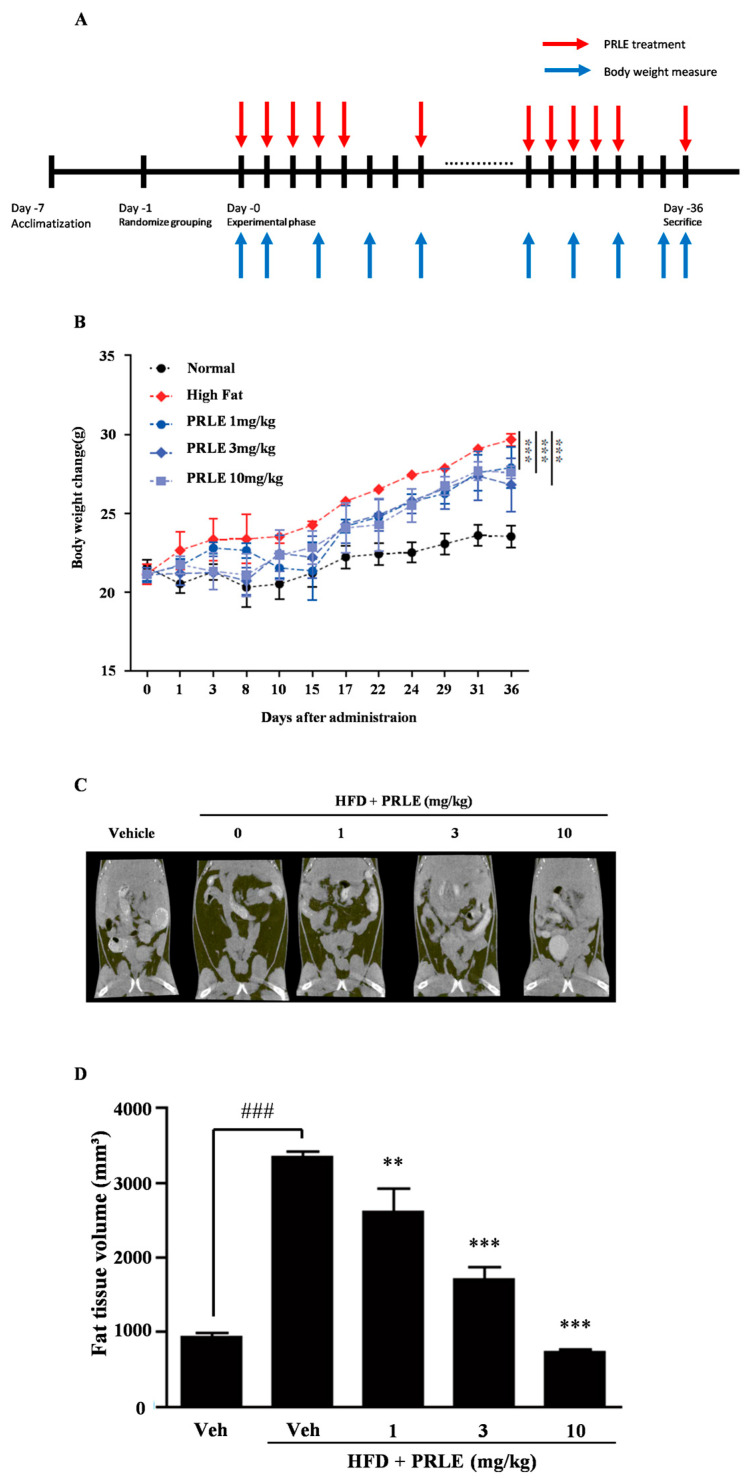
**PRLE administration reduced increases in body weight and body fat in HFD-fed mice**. (**A**) A scheme of animal experimental design. (**B**) The impact of PRLE treatment on body weight gain in HFD-fed mice over a period of 36 days is presented. Values are expressed as mean ± SD (*n* = 5). (**C**) Representative images of Micro-CT scans showing reduced whole-body fat in PRLE-treated HFD-fed mice. (**D**) The results of statistical analysis of μ-CT values of the PRLE-treated HFD-fed mice conducted 36 days after PRLE treatment are presented. Values are expressed as mean ± SD. ### *p* < 0.001 (compared to standard diet control), ** *p* < 0.01, *** *p* < 0.001 (compared to HFD control).

We used to examine the anti-obesity effects of PRLE in HFD-fed mice, a mouse model of obesity. The PRLE-treated HFD-fed mice showed a lower rate of weight gain than the PRLE-untreated HFD-fed mice (Figure 3B). To examine further the anti-obesity effects of PRLE in HFD-fed mice, we assessed whole-body fat accumulation using Micro-CT. The HFD group exhibited more fat accumulation than the standard diet group (Figure 3C). Conversely, the PRLE-treated HFD-fed mice displayed a reduction in body fat relative to the PRLE-untreated HFD-fed mice (Figure 3D). These findings indicate that PRLE exerts anti-adipogenic properties in an HFD-induced obesity mouse model.

### 2.4. Epididymal White Adipose Tissue (WAT) Is Diminished in HFD-Fed Mice

**Figure 4:**
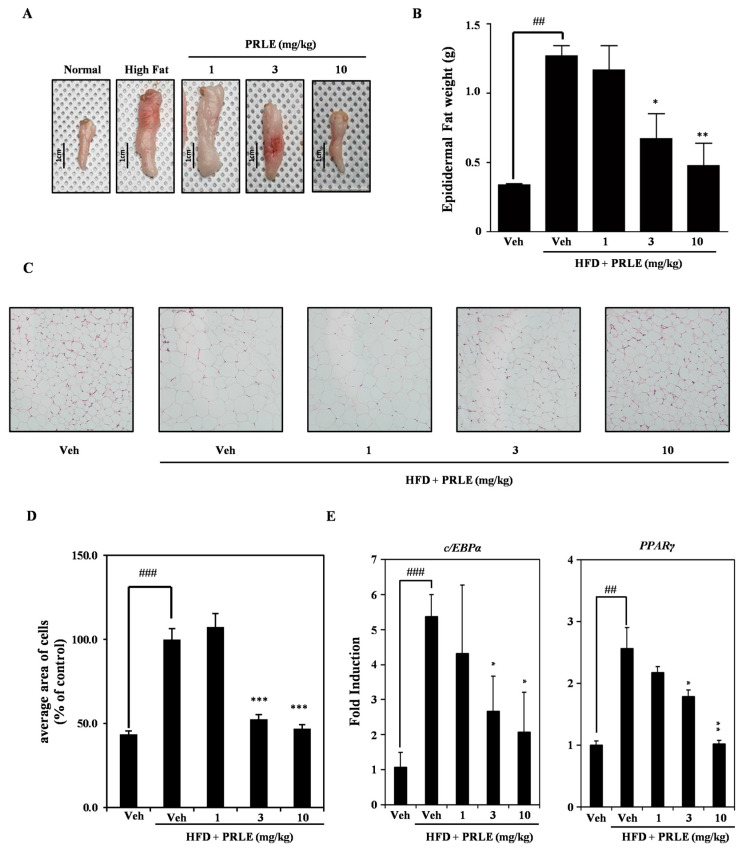
**The impact of PRLE on body fat accumulation in an HFD-fed mouse obesity model.** (**A**) Representative image of epididymal white adipose tissue in standard diet-fed mice and PRLE untreated and treated HDF-fed mice. (**B**) Quantitative analysis of epididymal white adipose tissue mass (*n* = 5). Statistical significance is indicated as ## *p* < 0.01 (compared to standard diet control), * *p* < 0.05; ** *p* < 0.01 (compared to HFD control). (**C**) Representative image of H&E staining of epididymal white adipose tissue sections post-PRLE treatment (Magnification: ×200). (**D**) Average adipocyte size following PRLE treatment. Statistical significance is indicated as ### *p* < 0.001 (compared to standard diet control), *** *p* < 0.001 (compared to HFD control). (**E**) After experiment period, the mRNA expression of transcription factors involved in adipocyte differentiation was assessed using qRT-PCR in epididymal WAT stimulated with either vehicle or PRLE at 1, 3, 10 mg/kg. β-actin served as the internal control. ## *p* < 0.01, ### *p* < 0.001 (compared to standard diet control), * *p* < 0.05, ** *p* < 0.01 (compared to HFD control).

Epididymal WAT was collected to evaluate the anti-obesity effects of PRLE in the context of a high-fat diet. PRLE significantly decreased both the volume (Figure 4A) and weight (Figure 4B) of epididymal WAT in HFD-fed mice. To assess adipocyte size, epididymal fat was collected and subjected to staining with hematoxylin and eosin. The results showed that the sizes of the adipocytes in the HFD-fed mice were smaller than those in the PRLE-treated HFD-fed mice (Figure 4C,D). To elucidate the molecular mechanisms underlying epididymal white adipose tissue, RNA was extracted from this tissue, and quantitative reverse transcription polymerase chain reaction (qRT-PCR) was conducted. The results indicated that PRLE inhibited C/EBP alpha and PPAR gamma in a dose-dependent manner (Figure 4E). Therefore, PRLE treatment had anti-obesity effects in an HFD-fed mouse model of obesity.

## 3. Discussion

In the current study, we demonstrated that *Paliurus ramosissimus* leaf extract (PRLE) treatment exhibits anti-obesity effects both in vitro and in vivo, with no detectable cytotoxic effects. This provides novel evidence supporting the anti-obesity potential of PRLE, which has been rarely investigated to date.

Anti-obesity pharmacological agents are generally classified into three categories: appetite suppressants, fat absorption inhibitors, and glucagon-like peptide 1 (GLP-1) analogs [13]. Appetite suppressants, such as phentermine and sibutramine, function by modulating neurotransmitters, including norepinephrine and serotonin, to decrease food intake [14,15]. Fat absorption inhibitors, such as orlistat, reduce the absorption of dietary fat in the intestines [16,17]. GLP-1 analogs, including liraglutide and semaglutide, target GLP-1 receptors to enhance satiety and diminish appetite [18,19]. Despite their efficacy, numerous anti-obesity medications have been withdrawn from the market due to their severe adverse effects. For example, amphetamines, rimonabant, and sibutramine were removed due to their detrimental impacts on cardiovascular health [20]. This situation has prompted increasing interest in natural products as potential anti-obesity agents. For instance, a recent study reported that the ethanol extract of Geranium wilfordii Maxim inhibited both adipogenesis and lipogenesis [21]. In line with such findings, our results are consistent with other studies showing that various plant extracts exert anti-obesity effects by regulating adipogenesis. For example, *Ziziphus jujuba* leaf extract was found to suppress adipocyte differentiation through the PI3K/AKT pathway [22], while *Ramulus mori* extract demonstrated both anti-adipogenic and anti-obesity effects in high-fat diet-induced obese mice [23]. Taken together, these results, along with our study, suggest that natural products, including members of the Rhamnaceae family, may exert anti-obesity activity by targeting common regulatory pathways.

The investigation of 3T3-L1 cell differentiation serves as a valuable method for screening potential treatments for obesity [24]. The complete maturation of adipocytes is characterized by the formation of lipid droplets, which can be visualized using Oil Red O staining. In this study, we induced 3T3-L1 pre-adipocytes to undergo adipocyte differentiation and subsequently assessed by Oil Red O staining [25]. The findings indicated that treatment with PRLE significantly hindered the accumulation of lipid droplets and reduced the number of Oil Red O-positive cells. These results suggest that PRLE effectively inhibits adipocyte differentiation.

Adipocyte differentiation is regulated by a diverse array of transcription factors, including C/EBPα and PPARγ. PPARγ is widely considered as a principal adipogenesis regulator and plays a crucial role in maintaining insulin sensitivity [26]. C/EBPα binds to the PPARγ promoter region, which subsequently enhances the expression of the PPARγ isoform 2, thereby promoting adipogenesis [27]. FABP4 is significantly expressed during adipocyte differentiation and is transcriptionally regulated by PPARγ [28]. Leptin, a peptide hormone, is instrumental in regulating food intake, body mass, and reproductive function, while it also contributes to pro-inflammatory immune responses, fetal growth, angiogenesis, and lipolysis [29]. In the present study, PRLE dose-dependently decreased mRNA expression of genes associated with adipogenesis, including PPARγ, C/EBPα, FABP4, and leptin. PPARγ and C/EBPα are essential for the conversion of pre-adipocytes into mature adipocytes [30]. C/EBPβ, a key transcription factor directing the differentiation process, is the first to be activated when pre-adipocytes are subjected to a differentiation medium [31]. Notably, PPARβ expression does not change significantly during the differentiation of 3T3-L1 cells [32]. Our data indicate that the protein expression levels of C/EBPα, C/EBPβ, PPARγ, and FABP4 were decreased dose-dependently by PRLE.

In adipose tissue, AMPK has different functions, including the facilitation of lipolysis, fatty acid oxidation, and glucose transport, while concurrently inhibiting lipogenesis, fatty acid synthesis, and the production of pro-inflammatory cytokines [33]. In humans, AMPK activity is lower in the WAT of individuals with insulin resistance, but it can increase following weight loss interventions [34]. Furthermore, ACC plays a pivotal role in lipid condensation and is essential for the overall regulation of energy metabolism [35]. In HFD-fed mice, ACC activation is observed, which subsequently promotes adipogenesis [36]. The phosphorylation of ACC inhibits its enzyme activity, which could be employed to reduce significantly lipid production and thereby prevent the onset of obesity [37]. Numerous studies have underscored the critical role of AKT in adipocyte differentiation. Additionally, the signaling pathway of phosphoinositide 3-kinase/AKT (PI3K/AKT) has been implicated in the anti-adipogenic effects of Ziziphus jujuba Mill. leaf extract and its isolated compounds [22,38]. In the present study, treatment with PRLE increased phosphorylation of both AMPK and ACC. Moreover, the MDI-induced phosphorylation of AKT was diminished following PRLE treatment. Together, the findings indicate that PRLE inhibits lipid accumulation, probably via its effects on the regulation of signaling pathways associated with adipogenesis.

Numerous mouse models of obesity have been established to develop new treatments for this health condition. The first model reported involved the administration of a HFD to mice, while the second model reported involved establishing mice deficient in leptin (ob/ob). However, the leptin-deficient model does not accurately replicate the complexities of obesity in humans. By contrast, the HFD-induced obesity mouse model provides a more accurate representation of human obesity [39]. Male mice are frequently chosen for obesity studies because they are more susceptible to high-fat diets, making them more prone to developing diet-induced insulin resistance and glucose intolerance. In contrast, female mice generally gain weight more slowly, have a lower incidence of obesity, and are typically more resistant to obesity caused by high-fat diets [40,41]. Consequently, we opted to utilize the HFD mouse model in this study. HFD resulted in significant body weight gain, which was subsequently inhibited by treatment with PRLE. Notably, PRLE-treated HFD-fed group mice exhibited reduced abdominal fat tissue levels. Epididymal WAT secretes various cytokines that play a crucial role in metabolic regulation during HFD-induced obesity [42]. Our findings indicated that both the size and weight of epididymal WAT decreased following PRLE treatment. The proliferation of epididymal WAT is observed at an early stage in mice subjected to diet-induced obesity [43]. Furthermore, the loss of mass in epididymal WAT is attributed to a reduction in both the size and number of adipocytes [44]. Additionally, our results demonstrated that a 5-week treatment regimen with PRLE effectively reduced the levels of epididymal WAT. The findings demonstrate that PRLE effectively mitigates obesity in an in vivo model that closely replicates this condition in humans. The mRNA expression levels of PPARγ and C/EBPα in epididymal WAT were observed to decrease following treatment with PRLE.

This study has several limitations that should be acknowledged. The specific bioactive compounds responsible for the observed anti-obesity effects of *Paliurus ramosissimus* leaf extract (PRLE) have not yet been identified. To address this limitation, ongoing experiments in our laboratory are focused on chemical characterization of the extract and additional functional assays to further elucidate its anti-obesity activity. Despite the extensive investigation of natural products for their anti-obesity potential, studies on *P. ramosissimus* remain very limited, and no previous report has described its inhibitory effects on adipogenesis. Importantly, our study provides comprehensive evidence by integrating both in vitro adipocyte differentiation assays and an in vivo high-fat diet-induced obesity model, thereby establishing consistent results across different biological systems. Furthermore, the anti-obesity effect of PRLE was mechanistically associated with the downregulation of key transcription factors, including PPARγ and C/EBPα, underscoring its distinct action through the inhibition of adipogenesis. Collectively, these findings suggest that PRLE inhibits adipocyte differentiation both in vitro and in vivo in high-fat diet-induced obesity through the suppression of PPARγ.

## 4. Materials and Methods

### 4.1. Preparation of PRLE

*Paliurus ramosissimus* leaves were collected in June and July 2020 from Jeju, South Korea. The species *Paliurus ramosissimus* was officially identified by Professor Gyu-Young Chung at Andong National University (voucher specimen: FMCPrJJ-1909-1-3). The leaves were thoroughly washed with clean, sterile water and subsequently air-dried at 50 °C for three days to eliminate moisture. Following this, the dried leaves of *Paliurus ramosissimus* were pulverized using a blender. A total of 20 g of the powdered material was extracted in a shaking incubator with 400 mL of 70% ethanol for 48 h at room temperature. After centrifugation of the crude extracts, the supernatant was filtered using Advantec filter paper No. 2 (Tokyo, Japan) and concentrated at 45 °C under reduced pressure in an evaporator to obtain a dry powder. The resulting PRLE was then reconstituted in 100 mg/mL dimethyl sulfoxide (DMSO) (Sigma-Aldrich, St. Louis, MO, USA). For animal administration, the PRLE dry powder was suspended in 0.5% carboxymethylcellulose (CMC) (Sigma-Aldrich, St. Louis, MO, USA) dissolved in phosphate-buffered saline (PBS) (Thermo Fisher Scientific, Waltham, MA, USA).

### 4.2. Reagents and Antibodies

Fetal bovine serum (FBS) and antibiotics, specifically streptomycin and penicillin, were purchased from Gibco (Thermo Fisher Scientific, Waltham, MA, USA). TRIzol was purchased from Invitrogen (Carlsbad, CA, USA). Isobutylmethylxanthine (IBMX), dexamethasone, and insulin were obtained from Sigma-Aldrich (St. Louis, MO, USA). Anti-actin and horseradish peroxidase (HRP)-conjugated secondary antibodies (for both rabbit and mouse) were from Santa Cruz Biotechnology (Santa Cruz, CA, USA). Additionally, antibodies targeting C/EBPα, PPARγ, FABP4, and ATF-3 were provided by Cell Signaling Technology (Beverly, MA, USA).

### 4.3. Cell Culture

Mouse 3T3-L1 pre-adipocytes, obtained from the American Type Culture Collection (ATCC CL-173™, Manassas, VA, USA), were cultured in Dulbecco’s Modified Eagle’s Medium (DMEM) supplemented with 10% FBS and antibiotics. The cultures were maintained at 37 °C in a 5% CO_2_ atmosphere in an incubator (MCO-170AIC-PK; Panasonic, Osaka, Japan), with culture medium replacement every 2–3 days. To induce differentiation, the immature adipocytes were grown in a medium containing 0.5 mM isobutylmethylxanthine (IBMX), 5 μM dexamethasone, 0.5 μg/mL insulin, and 10% FBS, with day 0 designated as the day differentiation was initiated. The medium was replaced with fresh medium every 48 h. After differentiation for 2 days, the cells were maintained in a medium containing 1 μg/mL insulin and 10% FBS, with medium changes at 2-day intervals for 8 days. The cells used in subsequent experiments were cells from passages 5–7.

### 4.4. Cell Viability Assay

PRLE cytotoxic effects on 3T3-L1 cells were assessed using the Cell Counting Kit-8 (CCK-8) assay. In brief, after seeding into 96-well plates, cells were cultured for 24 h in the presence of different concentrations of PRLE. The CCK-8 assay (Dojindo Molecular Technologies, Rockville, MD, USA) was used to subsequently assess cell viability. The culture supernatant was replaced with a new medium containing CCK-8 solution at a ratio of 9:1 for 30 min. A spectrophotometer (SpectraMax iD3; Molecular Devices, Sunnyvale, CA, USA) was then used to measure optical density at 450 nm.

### 4.5. Oil Red O Staining

Lipid accumulation was assessed by Oil Red O staining (Sigma-Aldrich, St. Louis, MO, USA). In brief, the cells were cultured in the presence of various PRLE concentrations for designated time periods. After incubation, the cells were rinsed twice with phosphate-buffered saline and then fixed in 10% formalin for 5 min, rinsed with distilled water, and stained for 30 min with 0.5% Oil Red O in 60% isopropanol. The stained lipid droplets within adipocytes were captured and imaged using an inverted microscope (Leica Microsystems, Wetzlar, Germany). The stained lipid droplets were then extracted using 100% isopropanol, and the samples were transferred to a 96-well plate for absorbance measurement at 510 nm using a spectrophotometer (SpectraMax iD3; Molecular Devices).

### 4.6. RNA Isolation and Reverse Transcription Quantitative Polymerase Chain Reaction (RT-qPCR)

mRNA expression levels in differentiated cells were assessed by RT-qPCR. TRIzol reagent (Invitrogen, Waltham, MA, USA) was used to isolate total RNA from cells or epididymal WAT according to the manufacturer’s recommendations. In brief, cells were lysed with an appropriate volume of TRIzol reagent following a wash with phosphate-buffered saline (PBS). For epididymal WAT, tissues were chopped with scissors, homogenized, and lysed by adding an appropriate volume of TRIzol reagent. After chloroform addition and incubation on ice for 10 min, the lysates were subjected to centrifugation at 15,000 rpm for 10 min at 4 °C. The supernatants were collected and placed in a new tube. After 2-propanol addition, the crude RNA was subjected to centrifugation again at 15,000 rpm for 10 min at 4 °C. After washing the RNA pellets in 75% ethanol in DEPC water, RNA was quantified using a NanoDrop™ 2000 spectrophotometer (Thermo Scientific, Waltham, MA, USA). Subsequently, the RevertAid First Strand cDNA Synthesis Kit (Thermo Scientific, Waltham, MA, USA), was used to reverse-transcribe 1 μg of total RNA according to the manufacturer’s instructions. Primer design was performed using the online Primer3 software (ver. 2.5.0) [45] and the primer sequences are shown in Table 1. QuantStudio™ 5 real-time PCR System (Thermo Scientific, Waltham, MA, USA) and PowerUp™ SYBR™ Green Master Mix (Thermo Scientific, Waltham, MA, USA) were used to perform SYBR Green-based RT-qPCR. All samples were analyzed in triplicate, and data processing was performed using the 2^−ΔΔCT^ method [46]. *HPRT1* served as the internal control.

### 4.7. Western Blotting

RIPA lysis buffer (Cell Signaling Technology, Danvers, MA, USA) containing a protease inhibitor was used to extract total protein from 3T3-L1 cells. Following incubation for 10 min on ice, the lysates were centrifuged at 15,000 rpm for 15 min. Detergent-Compatible (DC) Protein Assay Kit (Bio-Rad, Hercules, CA, USA) was used to determine the protein concentration of the lysates. Each sample (30 µg protein) was loaded onto a 7.5–15% sodium dodecyl sulfate-polyacrylamide gel, subjected to electrophoresis, and the proteins were transferred to polyvinylidene fluoride membranes (Merck Millipore, Darmstadt, Germany). The membranes were incubated with primary antibodies followed by HRP-conjugated secondary antibodies. The primary and HRP-conjugated secondary antibodies were diluted 1:1000 and 1:3000, respectively, in 5% skim milk in TBST. Clarity Western ECL Substrate (Bio-Rad, Hercules, CA, USA) was employed for color development, and bands were visualized using a ChemiDoc XRS+ imaging system (Bio-Rad, Hercules, CA, USA).

### 4.8. In Vivo High-Fat Diet Obesity Model

This study followed the guidelines of the Standard Protocol for Animal Studies of the Department of Laboratory Animal Resources at the Yonsei Hospital Biomedical Research Institute. The Institutional Animal Care and Use Committee (IACUC) of Yonsei Hospital Biomedical Research Institute approved the experimental protocol (Permit No. 2021-0183). All measures were taken to minimize suffering, stress, and discomfort, as well as to keep the number of animals used to a minimum. Six-week-old male C57BL/6 mice, obtained from ORIENT BIO (Seongnam, Korea), were acclimatized for one week under a 12 h light/dark cycle, humidity levels of 50–60%, and a temperature of 22–24 °C. The mice were fed a standard laboratory diet with free access to water. Control mice were provided with a standard diet, in which the fat content provided 13.2% of the energy (3.02 kcal/g with 24% energy derived from protein, 13% from fat, and 62% from carbohydrate; LabDiet 5053; LabDiet, St. Louis, MO, USA). Obesity was induced by feeding the mice a high-fat diet (HFD; 5.24 kcal/g with 20% energy derived from protein, 60% from fat, and 20% from carbohydrate; D12492; Research Diets, New Brunswick, NJ, USA), the fat content of which provided 60% of the energy. The mice were randomly assigned to the following groups (*n* = 5 mice per group): vehicle (standard diet + water), HFD (HFD + water), and treatment groups receiving 10, 50, and 100 mg per kg of PRLE (HFD + PRLE). The administrated agents were dissolved in PBS containing 0.5% carboxymethylcellulose (CMC). Mouse body weight was recorded every two days. The treatments (100 µL) were administered five times a week for a duration of 36 days via intragastric gavage using a feeding needle, without the use of anesthesia. No blinding was performed at any stage of this study.

### 4.9. ARRIVE Guidelines

A pre-clinical study was designed as a prospective and randomized trial to assess the anti-obesity effects of PRLE. The sample size for each group was determined using G-power software (ver. 3.1.9.7) based on preliminary studies. It was concluded that three mice per group were sufficient to evaluate the effects of HPWE, with an additional two mice included to account for potential unexpected mortality. However, no mortality was observed during the experimental period. The allocation of animals to treatment groups was conducted using a computer-generated randomization tool (https://www.randomizer.org/; accessed on 1 December 2021). Each animal was assigned a unique identification number, and cages were numbered according to their position on the rack. The following parameters were evaluated: body weight, micro-CT, and tissue extraction.

### 4.10. Morphological Analysis of Adipose Tissue Samples

Following the administration of food and pharmacological agents, the mice were anesthetized with Alfaxan (Jurox Inc., Kansas City, MO, USA) and subsequently euthanized through cervical dislocation. The epididymal white adipose tissue (WAT) was excised and weighed utilizing an electronic balance. Photographs of the epididymal WAT were taken with a digital camera, equipped with a 30 cm scale ruler for reference.

### 4.11. Micro-Computed Tomography (Micro-CT)

Abdominal fat volume was assessed utilizing an in vivo micro-tomography system (In vivo Micro-CT, Skyscan 1276, SKYSCAN N.V., Kontich, Belgium) one day prior to the conclusion of the experiment. The gray-scale intensity of the tissues within the mouse abdomen was quantified using the CtAn program (Bruker-Micro-CT Analyzer, Kontich, Belgium). Based on the intensity measurements obtained through the threshold method, the regions were categorized into lumbar vertebrae, lean tissue, adipose tissue, and skin. The extracted abdominal adipose tissue was reconstructed into a three-dimensional structure, which was subsequently employed for volume measurement. A threshold value of 20 was established, and the imaging process involved the removal of bone and muscle tissues, resulting in an image that exclusively captured the adipose tissue.

### 4.12. Hematoxylin and Eosin Staining

The isolated epididymal WAT samples were fixed in 10% formalin for a minimum duration of 24 h, subsequently embedded in paraffin, and sectioned with a sectioning machine (Leica Biosystems, Barrington, IL, USA) into 4-μm-thick slices. Following this, hematoxylin and eosin staining was conducted, and images were captured using an Aperio AT2 slide scanner (Leica Biosystems, Barrington, IL, USA). The size of the adipocytes was then quantified using ImageJ software (ver. 1.53k).

### 4.13. Statistical Analysis

All data are expressed as the mean ± standard deviation. Each experiment comprised three replicates for each variable and was conducted 3–5 times. Figure 1, Figure 2, Figure 3 and Figure 4 illustrate the results of representative experiments. Statistical significance was assessed using Student’s *t*-test and one-way analysis of variance (ANOVA), followed by Tukey’s honest significant difference post hoc test, utilizing the professional statistical software package GraphPad Prism 5.0 (GraphPad Software, San Diego, CA, USA). The significance level of *p* < 0.05 was established.

## 5. Conclusions

The present study demonstrates that PRLE treatment significantly reduces lipid accumulation in pre-adipocytes. Furthermore, PRLE treatment inhibited the expression of transcription factors associated with adipogenesis and decreased the levels of adipogenesis-related molecular markers at both the transcriptional and protein levels. Animal experiments indicated that PRLE effectively reduced body weight gain in HFD-fed mice, leading to a small weight of epididymal WAT, abdominal fat tissue, and the size of adipocytes within the epididymal WAT. Further work will be required to identify the specific compounds within the extract that contribute to its anti-obesity effects. Finally, our findings support the potential of PRLE as a therapeutic agent for the prevention of obesity.

## Figures and Tables

**Table 1:** Primer sequences.

Target Gene	Forward Primer (5′–3′)	Reverse Primer (5′–3′)
*C/EBPα*	GAA CAG CAA CGA GTA CCG GGT	GCC ATG GCC TTG ACC AAG GAG
*PPARγ*	CCA GAG CAT GGT GCC TTC GCT	CAG CAA CCA TTG GGT CAG CTC
*FABP4*	GGA TGG AAA GTC GAC CAC AA	TGG AAG TCA CGC CTT TCA TA
*Leptin*	CCT CAT CAA GAC CAT TGT CAC C	TCT CCA GGT CAT TGG CTA TCT G
*HPRT1*	TGC TCG AGA TGT CAT GAA GG	AGA GGT CCT TTT CAC CAG CA

## Data Availability

The original contributions presented in this study are included in the article/Supplementary Materials. Further inquiries can be directed to the corresponding author.

## Supplementary Materials

The following supporting information can be downloaded at: https://www.mdpi.com/article/10.3390/ph18101515/s1, PRLE inhibits adipogenic transcription factors and modulates signaling pathways in 3T3-L1 cells.

## Abbreviations

The following abbreviations are used in this manuscript:

GLP-1	glucagon-like peptide 1
HFD	high-fat diet
Micro-CT	Micro-Computed Tomography
MDI	a cocktail of methylisobutylxanthine, dexamethasone, and insulin
PRLE	*Paliurus ramosissimus* leaf extract
RT-qPCR	Reverse Transcription Quantitative Polymerase Chain Reaction
