# Supplementary figures and images for: Paliurus ramosissimus Leaf Extract Inhibits Adipocyte Differentiation In Vitro and In Vivo High-Fat Diet-Induced Obesity Through PPARγ Suppression
Source: Pharmaceuticals (Basel). 2025 Oct 10;18(10):1515. doi: 10.3390/ph18101515 (PMC12566692; doi:10.3390/ph18101515)

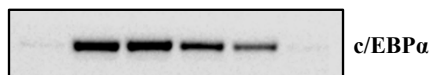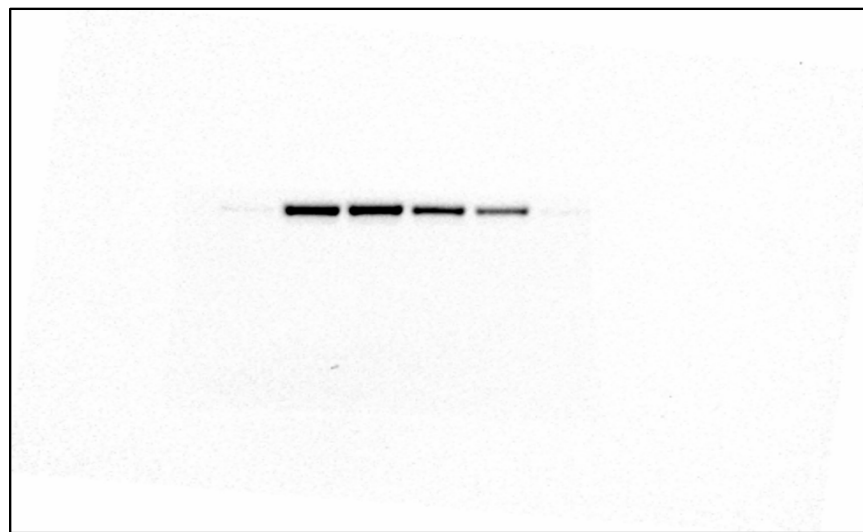

**c/EBP $\alpha$  (45)**

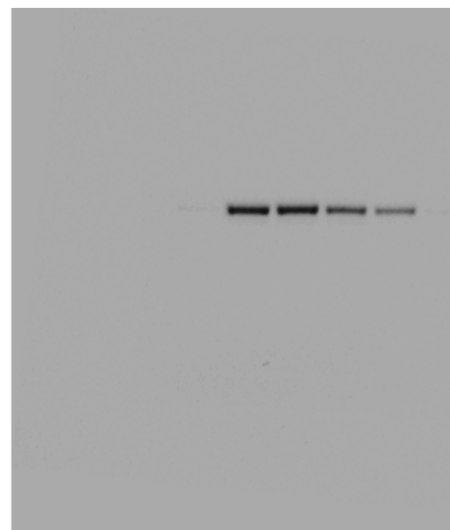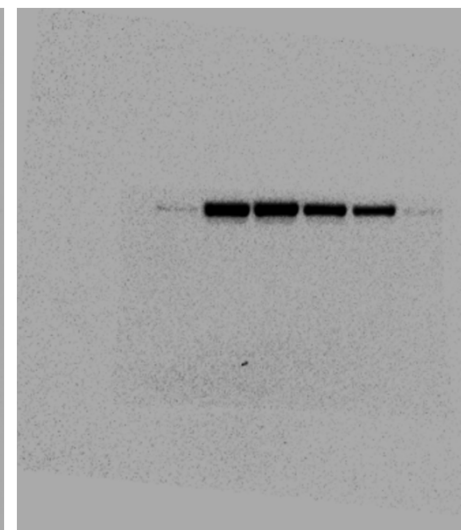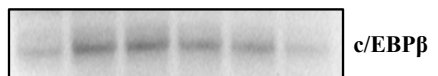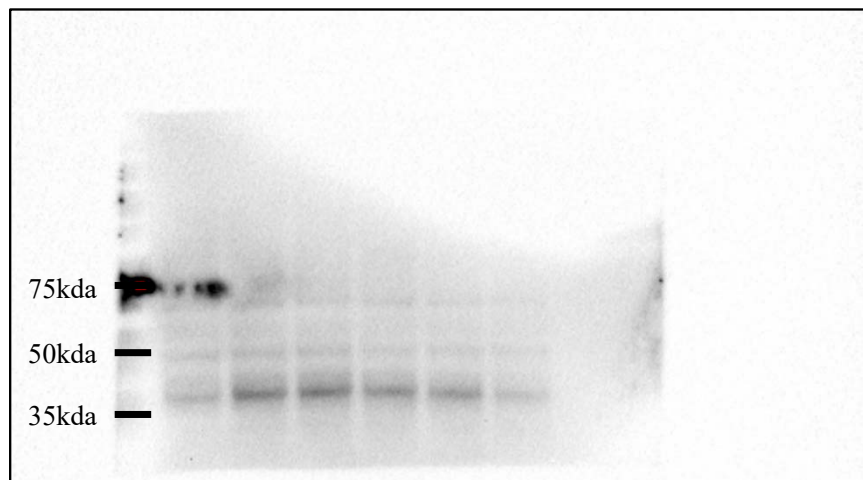

**c/EBP $\beta$  (50-33)**

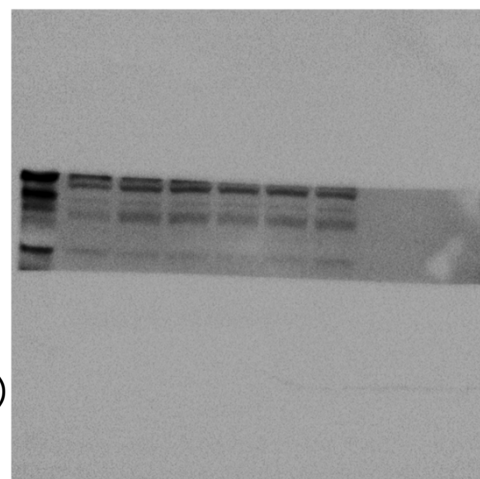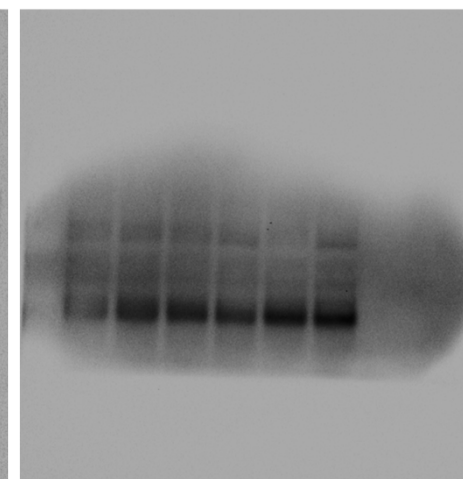

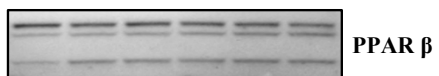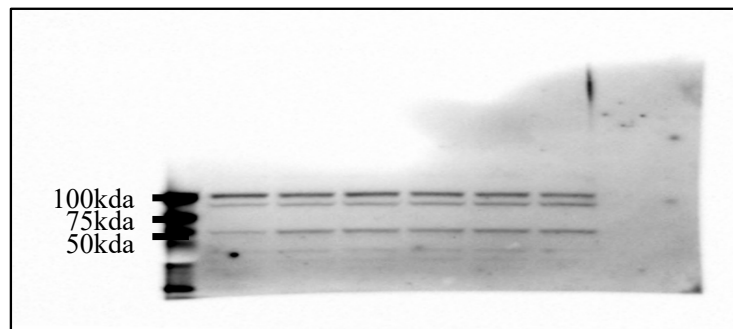

PPAR $\beta$  (51-90)

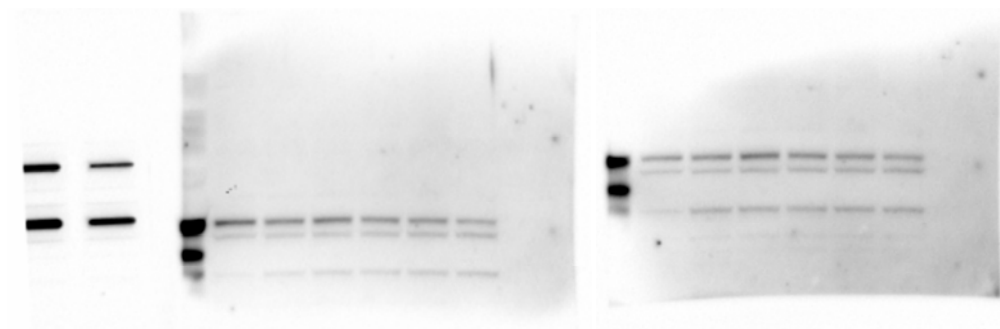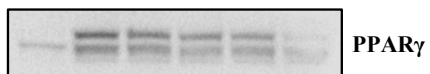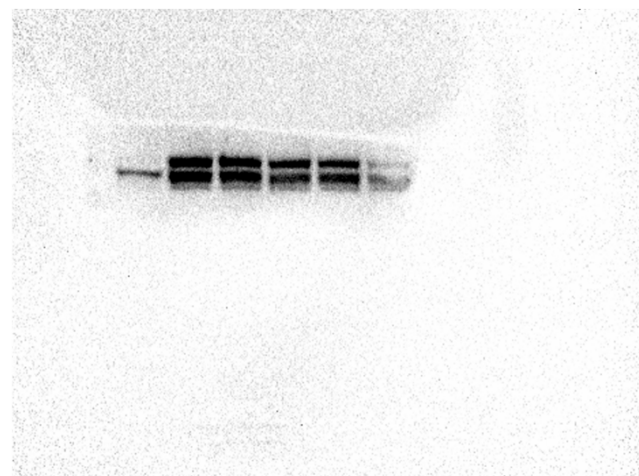

PPAR $\gamma$  (52)

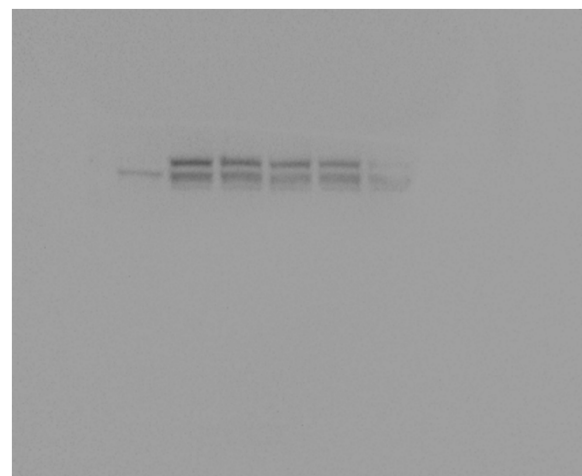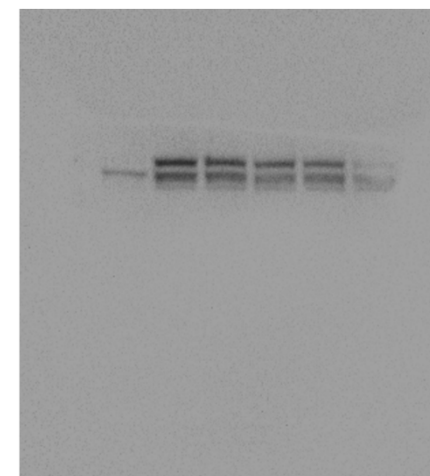

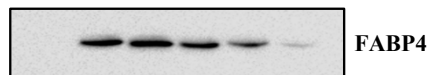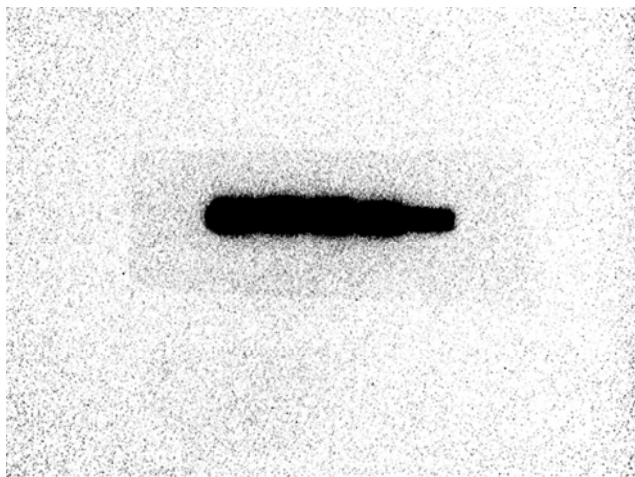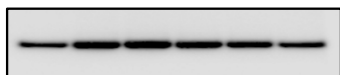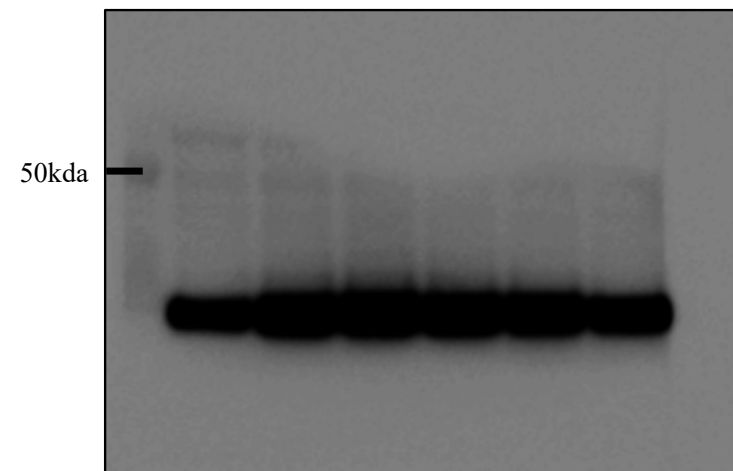

FABP4 (15)

HPRT1

HPRT1(30)

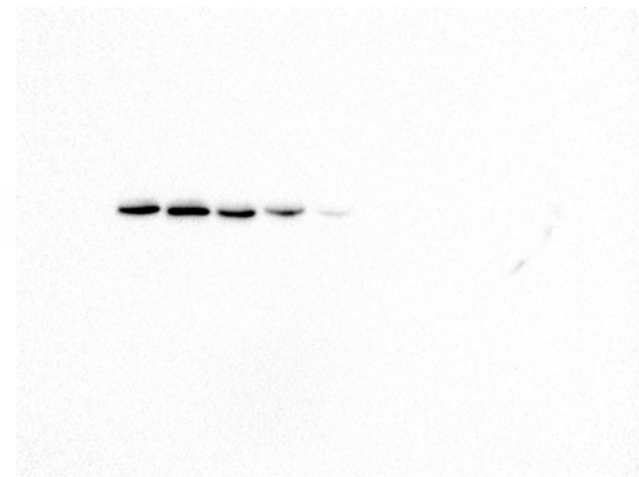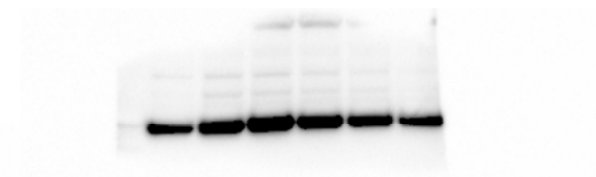

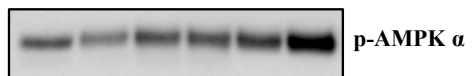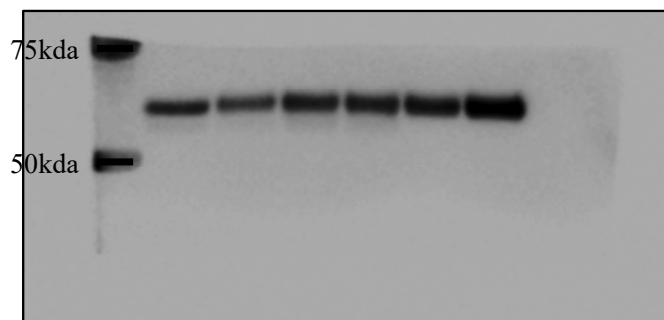

**p-AMPK α(62)**

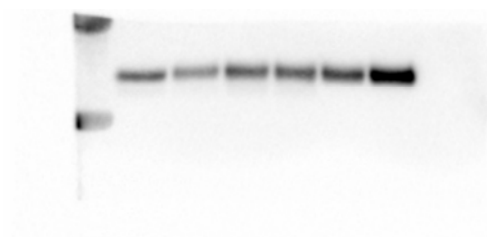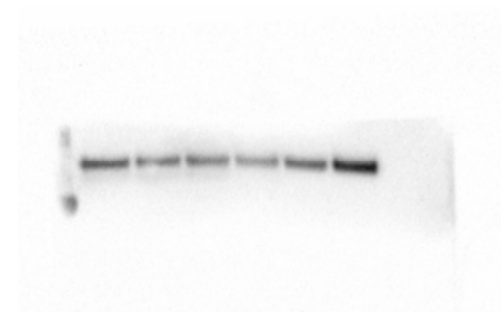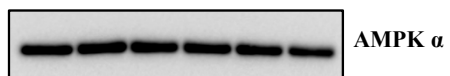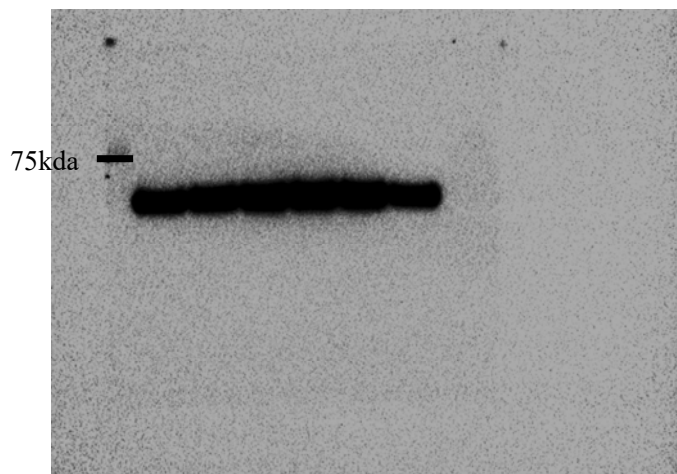

**AMPK α (62)**

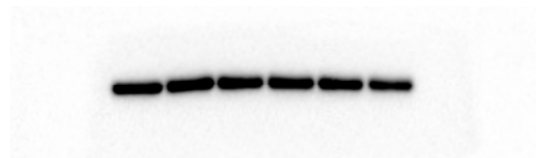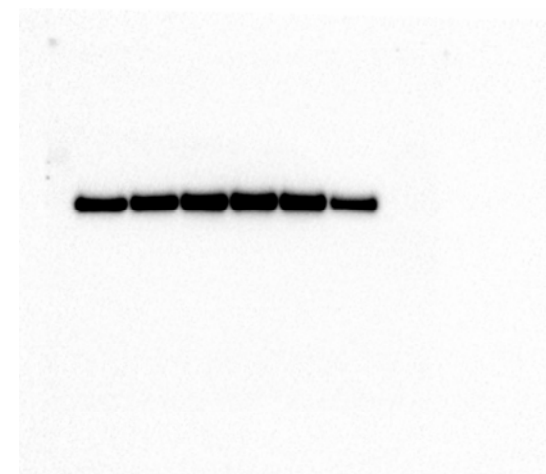

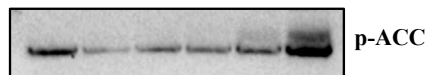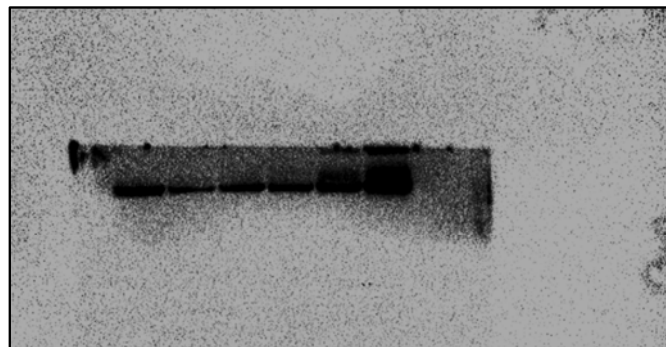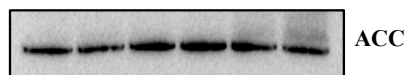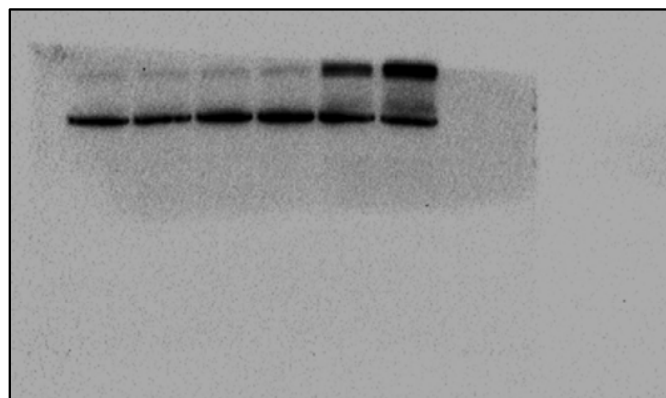

**p-ACC(280)**

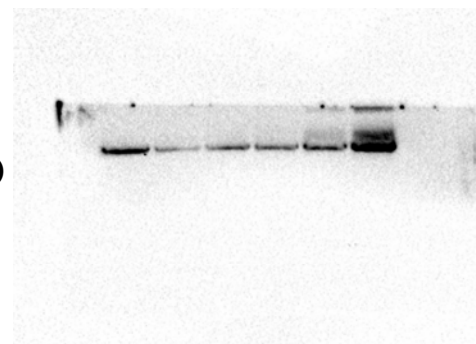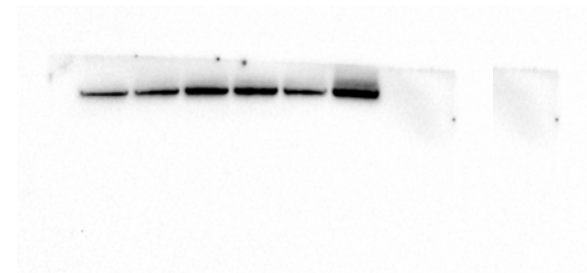

**ACC (280)**

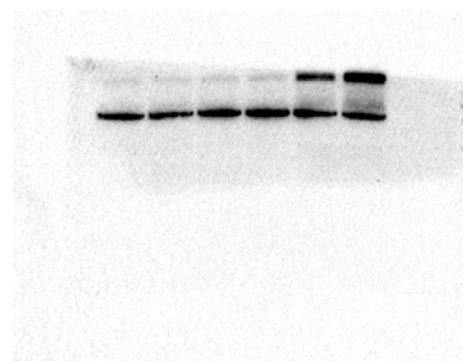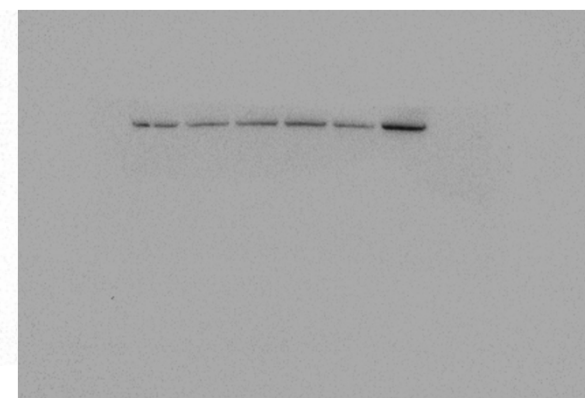

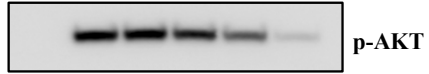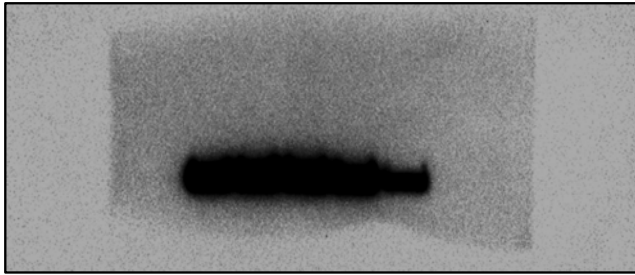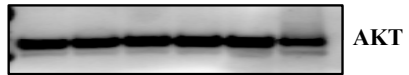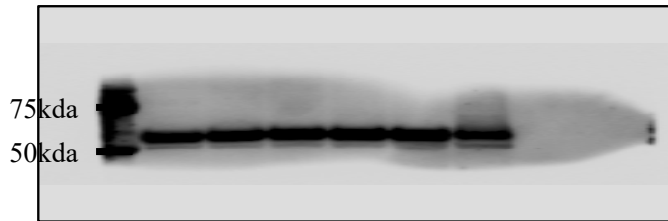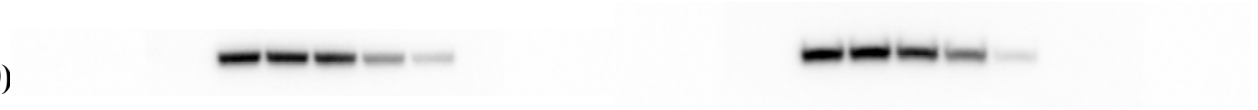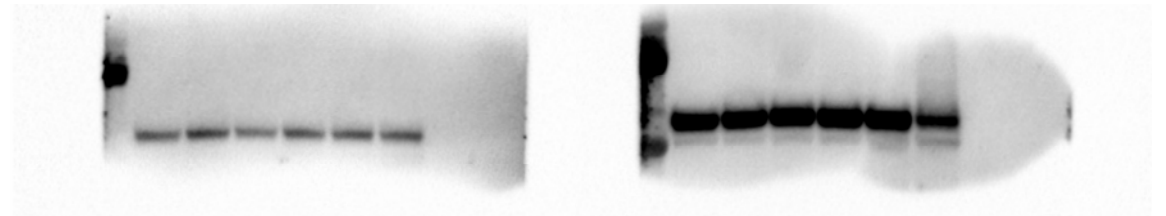

Supplement: Supplementary file 1 [file pharmaceuticals-18-01515-s001.zip › pharmaceuticals-3892769-supplementary.pdf]
